# The Epidemiological and Economic Impact of a Potential Foot-and-Mouth Disease Outbreak in Austria

**DOI:** 10.3389/fvets.2020.594753

**Published:** 2021-01-13

**Authors:** Tatiana Marschik, Ian Kopacka, Simon Stockreiter, Friedrich Schmoll, Jörg Hiesel, Andrea Höflechner-Pöltl, Annemarie Käsbohrer, Beate Pinior

**Affiliations:** ^1^Unit of Veterinary Public Health and Epidemiology, Institute of Food Safety, Food Technology and Veterinary Public Health, University of Veterinary Medicine Vienna, Vienna, Austria; ^2^Division for Animal Health, Austrian Agency for Health and Food Safety (AGES), Mödling, Austria; ^3^Division for Data, Statistics and Risk Assessment, Austrian Agency for Health and Food Safety (AGES), Graz, Austria; ^4^Department for Animal Health and Animal Disease Control, Federal Ministry of Labor, Social Affairs, Health and Consumer Protection, Vienna, Austria; ^5^Department of Veterinary Administration, Styrian Provincial Government, Graz, Austria

**Keywords:** control strategies, disease spread, economic consequences, EuFMDiS simulation model, foot-and-mouth disease

## Abstract

An outbreak of foot-and mouth disease (FMD) in an FMD-free country such as Austria would likely have serious consequences for the national livestock sector and economy. The objective of this study was to analyse the epidemiological and economic impact of an FMD outbreak in Austria in order to (i) evaluate the effectiveness of different control measures in two Austrian regions with different livestock structure and density, (ii) analyse the associated costs of the control measures and the losses resulting from trade restrictions on livestock and livestock products and (iii) assess the resources that would be required to control the FMD outbreak. The European Foot-and-Mouth Disease Spread Model (EuFMDiS) was used to simulate a potential FMD outbreak. Based on the epidemiological outputs of the model, the economic impact of the outbreak was assessed. The analysis of the simulations showed that the success of control strategies depends largely on the type of control measures, the geographical location, the availability of sufficient resources, and the speed of intervention. The comparison of different control strategies suggested that from an economic point of view the implementation of additional control measures, such as pre-emptive depopulation of susceptible herds, would be efficient if the epidemic started in an area with high livestock density. Depending on the chosen control measures and the affected region, the majority of the total costs would be attributable to export losses (e.g., each day of an FMD epidemic costs Austria € 9–16 million). Our analysis indicated that the currently estimated resources for surveillance, cleaning, and disinfection during an FMD outbreak in Austria would be insufficient, which would lead to an extended epidemic control duration. We have shown that the control of an FMD outbreak can be improved by implementing a contingency strategy adapted to the affected region and by placing particular focus on an optimal resource allocation and rapid detection of the disease in Austria. The model results can assist veterinary authorities in planning resources and implementing cost-effective control measures for future outbreaks of highly contagious viral diseases.

## Introduction

Foot-and-mouth disease (FMD) is a highly contagious viral disease affecting cloven-hoofed animals, which is known to spread rapidly within and between herds (1–5). An epidemic of FMD may have serious economic and social consequences on the livestock industry in affected countries, as demonstrated by a number of FMD outbreaks over the last 20 years (4–7). The FMD epidemic in the United Kingdom (UK), Ireland, The Netherlands and France in 2001 was one of the costliest livestock disease outbreaks reported. It was estimated that in the UK, which was the most severely affected, the outbreak resulted in losses of ~€ 5 billion, while a smaller outbreak in 2007 cost the public and private sectors € 146 million and € 68 million, respectively (8, 9).

The FMD outbreak in the UK was reported on 21 February 2001. Despite the immediate preventive measures in other European countries, epidemics of FMD were reported in France, Ireland and the Netherlands within a month (4). The Netherlands was the worst affected country outside the UK, with 26 infected farms, followed by France (2 infected farms) and Ireland (1 infected farm) (4, 7). The outbreak in the UK lasted for 214 days and resulted in the infection on 2,026 farms (10, 11). In total, 4 million animals were slaughtered for the purposes of disease control, with at least a further 2.5 million animals destroyed in welfare culls (11). To get the outbreak under control, The Netherlands decided to implement an emergency vaccination strategy. All susceptible animals on 1,800 farms in the affected region were vaccinated and these farms were subsequently depopulated. In total, ~260,000 animals were killed (4). During this crisis, different simulation models were used in attempt to predict the disease dynamics in the affected countries. This was one of the first time that models had been used during an outbreak to support veterinary authorities in their decision-making process (12). Simulation models allow exploration of various management strategies and provide frameworks that allow users to conceptualize and communicate their perceptions about the system (13). Models are especially useful as support tools for outbreak response planning in countries that have not (recently) experienced FMD outbreaks (1, 3, 14–20). The last FMD outbreak in the European Union (EU) occurred in Bulgaria in 2011, whereas in other countries, such as in Austria, the last FMD outbreak goes back several decades further, i.e., 1981 (21). Thus, there is a lack of knowledge about the extent of the spread of FMD with the current geographical distribution of farms, dynamics of livestock movements and availability of resources to effectively minimize the spread and associated economic consequences of an FMD outbreak in these countries (22).

Disease spread simulation models range from simple deterministic mathematical models (23), to complex spatially-explicit stochastic microsimulations (24, 25) some of which contain economic elements (26). While simple mathematical models can provide useful observations of disease behavior and outbreak dynamics, they tend to ignore the spatial, environmental, and social dimensions of epidemiology (27). Complex spatially-explicit simulation models with data-driven, individual-based modeling approach such as EuFMDiS (26), Australian Animal Disease Spread model (AADIS) (24), AusSpread (28), and Interspread Plus (25), while having higher data demands, are far more flexible and thus able to capture intricate regionalized spread dynamics. The aim of the study presented here was to analyse the epidemiological and economic impact of an FMD outbreak in Austria in order to (i) evaluate the effectiveness of various control measures against an FMD epidemic in two Austrian regions with different livestock structure and density; (ii) analyse the associated cost of the control measures and losses that arise from trade restrictions on livestock and livestock products; and (iii) assess the resources that would be required to control an FMD outbreak.

## Materials and Methods

### Description of the EuFMDiS Model

To simulate the hypothetical spread of FMD within and between herds in Austria, we used the European Foot-and-Mouth Disease Spread Model (EuFMDiS) (26) which is an European Union multi-country adaptation of the Australian Animal Disease Spread Model (AADIS) (24). EuFMDiS has a hybrid model architecture that combines equation-based modeling (used for simulating the spread of disease within a herd) with agent-based modeling (used for simulating the spread between herds). The spread of FMD virus between herds is one of the main processes within the model and it is simulated through five different pathways: (i) direct contact, (ii) indirect contact, (iii) local spread, (iv) airborne transmission, and (v) assembly centers (not used in this study). Each spread mechanism stochastically determines on any given simulation day whether disease is transmitted from an infected herd to a susceptible herd. Spread events through (i) direct contacts between herds are stochastically generated on a daily basis using Austrian movement data for the year 2018, which were obtained from the Austrian Animal Husbandry Register (Verbrauchergesundheitsinformationssystem, VIS). The probability of a consignment leaving an infected herd is determined from evaluated movement frequencies that depend on the herd type, region and season. The destination of the consignment (another herd, a slaughterhouse, a market, or export), is determined, based on the herd type and region. (ii) Indirect contacts between herds incorporate the spread of the virus due to farm visits of veterinarians, sharing of equipment between neighbors, milk tankers, and feed delivery vehicles. If a herd is exposed through indirect contact, the probability of transmission depends on the infectious prevalence of the source herd, the relative infectiousness of the source herd (based on species and herd size), environmental conditions that influence virus survival, biosecurity practices, and relative susceptibility of the exposed herd (based on species and herd size). As there is limited data on actual indirect contacts, the associated contact rates were estimated based on veterinary public health authority opinions. (iii) The local spread is defined as a transmission of disease from an infected herd to a susceptible herd within a short distance (within a 3 km radius). This includes the spread of local aerosol, spread across fences, straying of stock, people or sharing of equipment between neighbors. (iv) Airborne spread of the FMD virus is determined by wind direction and speed, atmospheric stability, precipitation, and relative humidity. The probability of occurrence of these factors is based on monthly data, which were obtained from 6 weather stations located across Austria. For each simulation day, the weather station closest to each candidate infectious herd is queried as to whether conditions are suitable for airborne spread. For each herd that is deemed a potential source of airborne spread, a sector is constructed in the prevailing wind direction for the month, subtended by a configurable angle of default size 30°. Topographical features such as mountains, lakes and forests that might influence the airborne spread are not considered in the model (26).

EuFMDiS simulates the disease spread under consideration of the control and eradication measures according to official policies defined in the EU Council Directive 2003/85/EG (29) and incorporates seven independent and concurrent control measures: (i) detection of first infected farm, (ii) movement restrictions, (iii) reporting of suspected farms, (iv) surveillance visits, (v) tracing, (vi) operation activities in infected farms (i.e., culling, disposal, cleaning, and disinfection), with options of pre-emptive culling of dangerous contact farms (categorized as potentially infected based on tracing high risk movements of livestock and its products) or pre-emptive depopulation of susceptible farms, (vii) vaccination [i.e., suppressive ring vaccination (i.e., carried out inside known infected areas in order to suppress virus shedding; it is accepted, however, that infection is probably present, and when time and resources permit, these animals will be slaughtered) or protective ring vaccination (i.e., carried out outside known infected areas in order to protect susceptible animals from infection) (30)]. These measures can be selected in the model in a range of different combinations. EuFMDiS assumes that surveillance activities, operation activities in infected farms, as well as vaccination will be carried out by “teams.” The number of teams is planned at the national level in accordance to available personnel, equipment, and consumables that are required to conduct each of the operational activities (Supplementary Table 1).

The epidemiological outputs of the model (i.e., epidemic duration, number of infected farms, number of farms culled, number of farms visited by surveillance teams etc.) are used to calculate the economic impact of the outbreak by consideration of the export losses and the cost of control activities (e.g., establishment of control centers, surveillance, culling and disposal of infected farms, cleaning and disinfection, vaccination, and compensation; Supplementary Table 2).

### FMD Spread and Control Strategies in Austria

Information on Austrian herds, including geographical locations, which were used in this study as input data for the model, was extracted from VIS for the period 2017–2018. The data included 5.32 million susceptible ruminants (51,014 cattle herds, 19,184 swine herds, 17,279 sheep and goat herds, and 19,190 backyard herds). For modeling purposes, the Austrian livestock population was categorized based on the type of species, herd size and production category, into nine farm types and eight herd types (Table 1).

**Table 1:** Herd and farm types data used in EuFMDiS model for the FMD outbreak simulations in Austria.

**Farm type^**a**^**	**Number of farms**	**Mean farm size (min–max)**	**Herd type^**b**^**	**Number of herds**	**Mean herd size (min–max)**
Large commercial dairy	5,799	85 (51–808)	Large commercial dairy	8,179	84 (51–808)
Large commercial beef	2,176	94 (51–1,481)	Large commercial beef	3,094	90 (51–1,481)
Small commercial beef	24,539	25 (10–50)	Small commercial beef	39,741	24 (1–50)
Commercial small ruminants	7,020	48 (10–3,221)	Commercial small ruminants	17,279	31 (1–3,221)
Large-scale commercial fattening pigs	1,876	707 (103–14,062)	Large-scale commercial fattening pigs	2,106	703 (103–14,062)
Large-scale commercial breeding pigs	634	785 (107–15,423)	Large-scale commercial breeding pigs	698	781 (107–15,423)
Small-scale commercial pig	3,078	171 (10–759)	Small-scale commercial pig	16,380	45 (1–759)
Backyard	19,190	5 (1–9)	Backyard	19,190	5 (1–9)
Mixed	19,770	62 (10–6,101)	–	–	–
Total	84,082			106,667	

N.B. The population of FMD-susceptible livestock is aggregated into farms of constituent herds based on species and farming practices.

a A farm can have one or more herds. A farm has static attributes (e.g., ID, type and constituent herd IDs), and dynamic attributes describing disease control and eradication status.

b *A herd has static attributes (e.g., ID, type, size, latitude and longitude, jurisdiction, region, and nearest weather station), and dynamic attributes describing infection status*.

**Figure 1:**
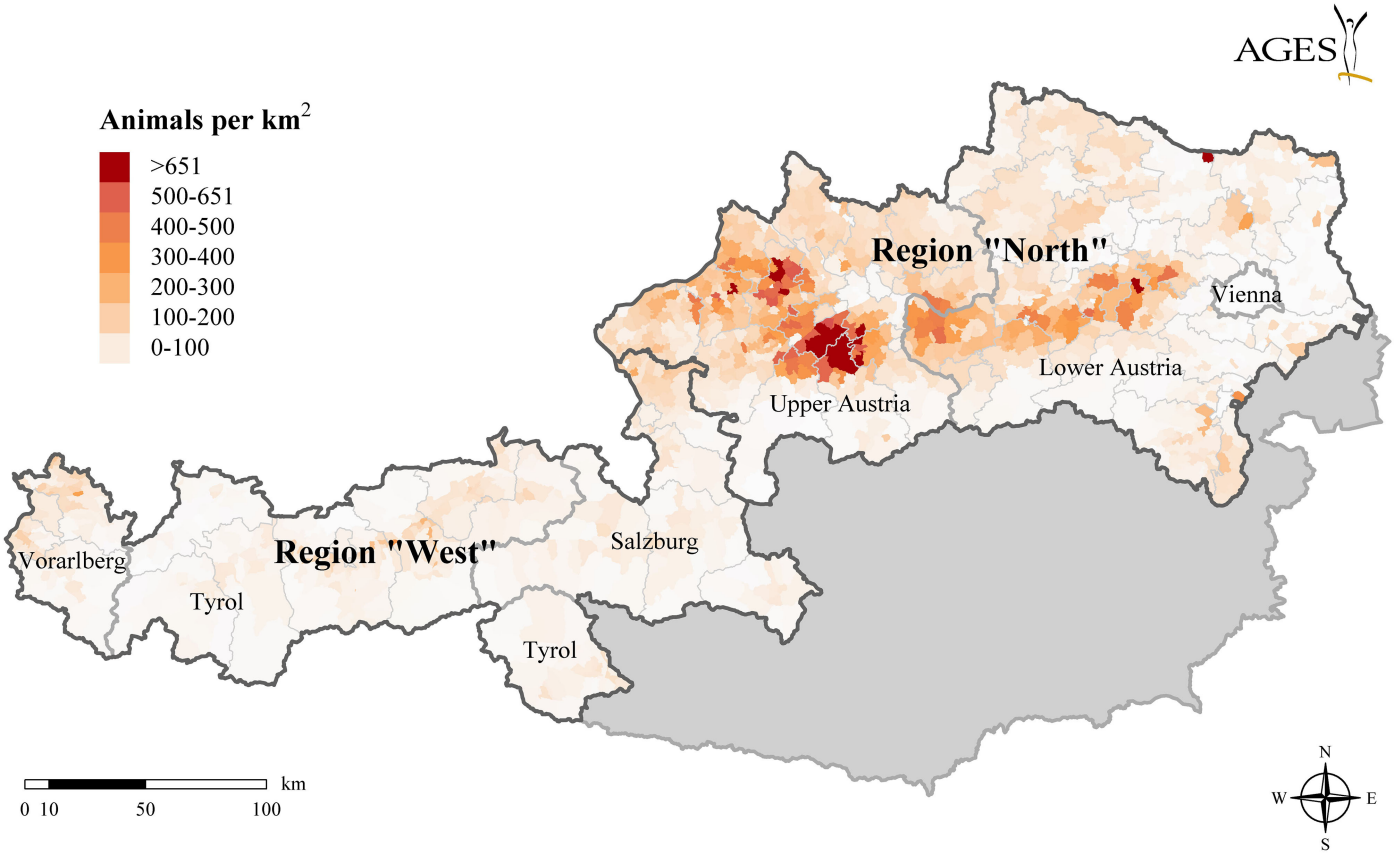
Map of the two study regions “*North*” and “*West*,” where the FMD outbreaks were initiated [Shapefiles source: Verwaltungsgrenzen (VGD) © BEV, 2019].

The spread of FMD was initiated in early autumn 2017 in two different regions (“*North*” and “*West*”) in Austria. Both regions differ with regard to livestock density, livestock production system, herd size, and herd type (Figure 1). Region “*North*” is a livestock-dense area (96 livestock animals/km^2^), which comprises mainly federal states Upper and Lower Austria and is characterized by an intensive livestock production (i.e., 58% of FMD-susceptible Austrian livestock population) and a high number of large cattle and swine herds [i.e., median (5th and 95th percentiles) of cattle herd size: 34 (10–115); median (5th and 95th percentiles) of swine herd size: 15 (1–801)], as well as a high rate of animal movements. Region “*West*” is situated mainly across the federal states of Tyrol, Vorarlberg, and Salzburg and is characterized by sparsely dense livestock areas, small herds [i.e., median (5th and 95th percentiles) of cattle herd size: 22 (8–75); median (5th and 95th percentiles) of swine herd size: 2 (1–27)] and numerous mountain pastures. The production is predominantly extensive and the density of susceptible livestock is low (26 animals/km^2^).

The FMD outbreak simulation was initiated in both regions in randomly chosen dairy cattle herds. In each iteration (1,000 per simulation) a different herd was chosen to consider the variation of herds and movement patterns in these regions. The same 1,000 index herds were used in all implemented control strategies and sensitivity analyses. The model results were presented as medians with the corresponding 5th and 95th percentiles. The outbreak was simulated to be undetected until day 21 (defined as silent phase), based on the experience from recent FMD epidemics (4, 31) and the natural behavior of the pathogen.

The implementation of control measures started after the silent phase (defined as control phase). The reference scenario included the following control measures according to the European (29) and national legislation (32): (i) 3 days national standstill on animal movements in the country, (ii) a three km radius zone (protection zone) around each infected herd in which movements between herds and out of the zone were restricted and herds were surveyed at least twice before lifting the zone, (iii) a 10 km radius zone (surveillance zone) around each infected herd in which movements between herds and out of the zone were restricted and herds were surveyed at least once before lifting the zone, (iv) culling, cleaning and disinfection of infected herds, and (v) tracing of movements from and to infected herds (Supplementary Figure 1). Supplementary Table 3 lists selected input parameters used in the simulation model.

In addition to the reference scenario, four alternative control scenarios based on variations of preventive depopulation and vaccination strategies were applied and are described in Table 2. The epidemiological and economic results of all alternative control strategies were compared to the reference scenario. The optimal control strategy was considered to be the strategy with the lowest total costs (direct plus indirect costs) of the outbreak. To examine the statistical differences in the outputs between various control strategies, we used the Wilcoxon rank sum test for paired observations. The analysis was performed in the statistical software XLSTAT (33), with the significance level set to < 0.05.

**Table 2:** Control strategies applied to control FMD outbreak in Austria using EuFMDiS.

**Control strategies**	**Description**
Reference scenario (SO)	Stamping out of all infected herds
Pre-emptive depopulation of dangerous contact herds (SODC)	Reference scenario and stamping out of dangerous contact herds, which were categorized as potentially infected based on tracing movements of livestock and its products
Pre-emptive depopulation of susceptible herds (SORC1)	Reference scenario and stamping out of all susceptible herds within a 1 km radius around infected herds, enforced 7 days after outbreak detection
Suppressive vaccination (SOSV1)	Stamping out of infected herds plus suppressive ring vaccination within 1 km radius of infected herds, enforced 7 days after outbreak detection
Protective vaccination (SOPV1)	Stamping out of infected herds plus protective ring vaccination within 1 km radius of infected herds, enforced 7 days after outbreak detection

### Costs and Losses of the FMD Outbreak in Austria

Direct costs included costs of control activities (including compensation payments) and were assessed by the EuFMDiS model (Supplementary Table 2). Indirect costs were estimated by our own economic model and included the following costs: losses due to export bans on livestock animal and livestock products (export losses), production losses for the farmers resulting from business interruption due to movement restrictions within protection and surveillance zones (production losses in zones) and losses resulting from temporary vacancy of stables for farmers, whose herds were culled (production losses in culled herds) (Supplementary Table 4). In the context of losses due to export bans, it is important to distinguish between export to EU and to non-EU countries and to consider different ban delays in scenarios according to implemented control measures. Thus, we estimated the export losses as follows: A total ban on livestock and livestock products to non-EU countries was assumed to last for 3 months after culling of the last infected animal and for 6 months in scenarios where protective vaccination was simulated. An extra 3-month delay, which represents the time until the OIE committee meets to declare Austria free from FMD was added to the ban period (14). For the intra-community trade (export to EU countries) we assumed, that the entire export of all live susceptible animals would be banned until the last infected animal was culled, including a 3 month delay and 6 month delay when protective vaccination was used. Losses due to a restriction on livestock product exports to EU countries were assumed to apply for the same period but only to farms in protection and surveillance zones (zoning), as suggested by Boklund et al. (14). The value of export losses in the affected region was estimated proportional to the regional production (14, 17). We assumed that during the FMD outbreak the Austrian imports and domestic consumption were unchanged in the short term. Production losses in zones were estimated for dairy cattle farms and resulted from not collecting the raw milk from dairy farms in surveillance and protection zones. Production losses in culled herds were assessed for all farm categories based on the contribution margin model, as suggested in the study by Waret-Szkuta et al. (34). The contribution margin for a farm (or single animal) equals the difference between the total revenue and total variable costs and so contributes to fixed cost coverage (34, 35). In case a detected herd was culled and there was subsequently no revenue from ongoing livestock activities, the contribution margin (per production category) represented the production losses for the time of business interruption (Supplementary Table 4).

### Sensitivity Analysis

Because input parameters in the epidemiological and economic modeling are subject to uncertainty, sensitivity analyses were conducted to assess the consequences of potentially uncertain inputs on the model outputs for both regions. A sensitivity analysis was performed for the following input parameters: (i) detection period of the first infected herd (−7 and +7 days), (ii) the length of national standstill (−3 and +3 days) and the maximum available resources, (iii) surveillance (+25 and +50%), and (iv) cleaning and disinfection (+25 and +50%). All other input parameters in the sensitivity analysis remained unchanged. The magnitude of the epidemic simulated for each of the sensitivity-scenarios was compared to the magnitude of the outbreak simulated for the reference scenario. Additionally, sensitivity analysis for cleaning and disinfection resources was conducted for pre-emptive depopulation scenario. These results were compared to the results of the depopulation control strategy prior to changing of the parameterization. Variations lower than 10% in the magnitude of the outbreaks through the changing of the input parameters were considered as evidence for the robustness of the model.

## Results

### Region “*North*”

**Figure 2:**
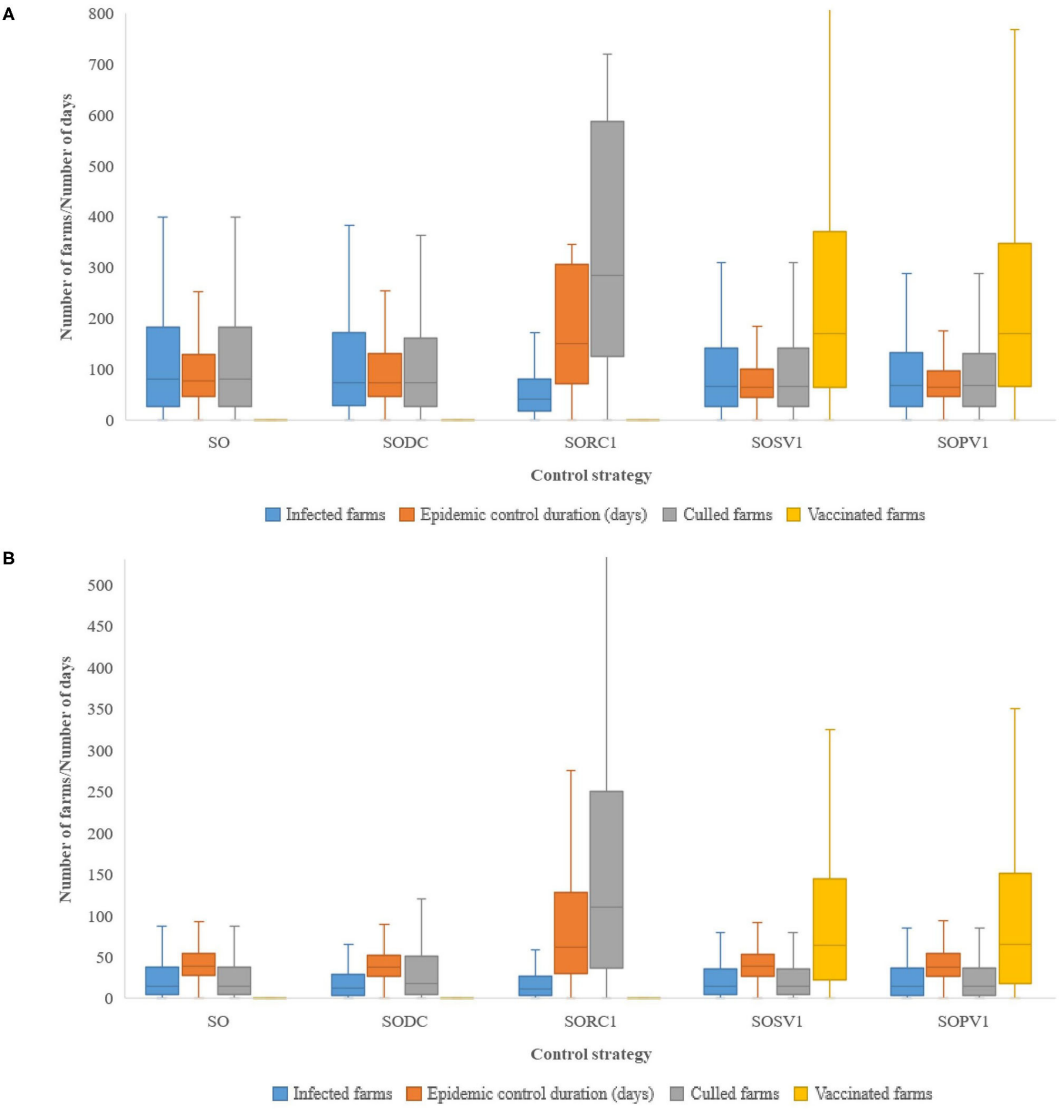
Box plots showing the epidemiological results of different control strategies for simulated FMD outbreaks initiated in **(A)** Region “*North*” and **(B)** Region “*West*.” The different control measures are: SO, stamping out of all infected herds (reference scenario); SODC, pre-emptive depopulation of dangerous contact herds; SORC1, pre-emptive depopulation of all susceptible herds within 1-km radius around infected herds; SOSV1, suppressive vaccination of all susceptible herds with 1-km radius around infected herds; SOPV1, protective vaccination of all susceptible herds with 1-km radius around infected herds. SODC, SORC1, SOSV1, and SOPV1 are the other additional control measures take into account in the model.

**Figure 3:**
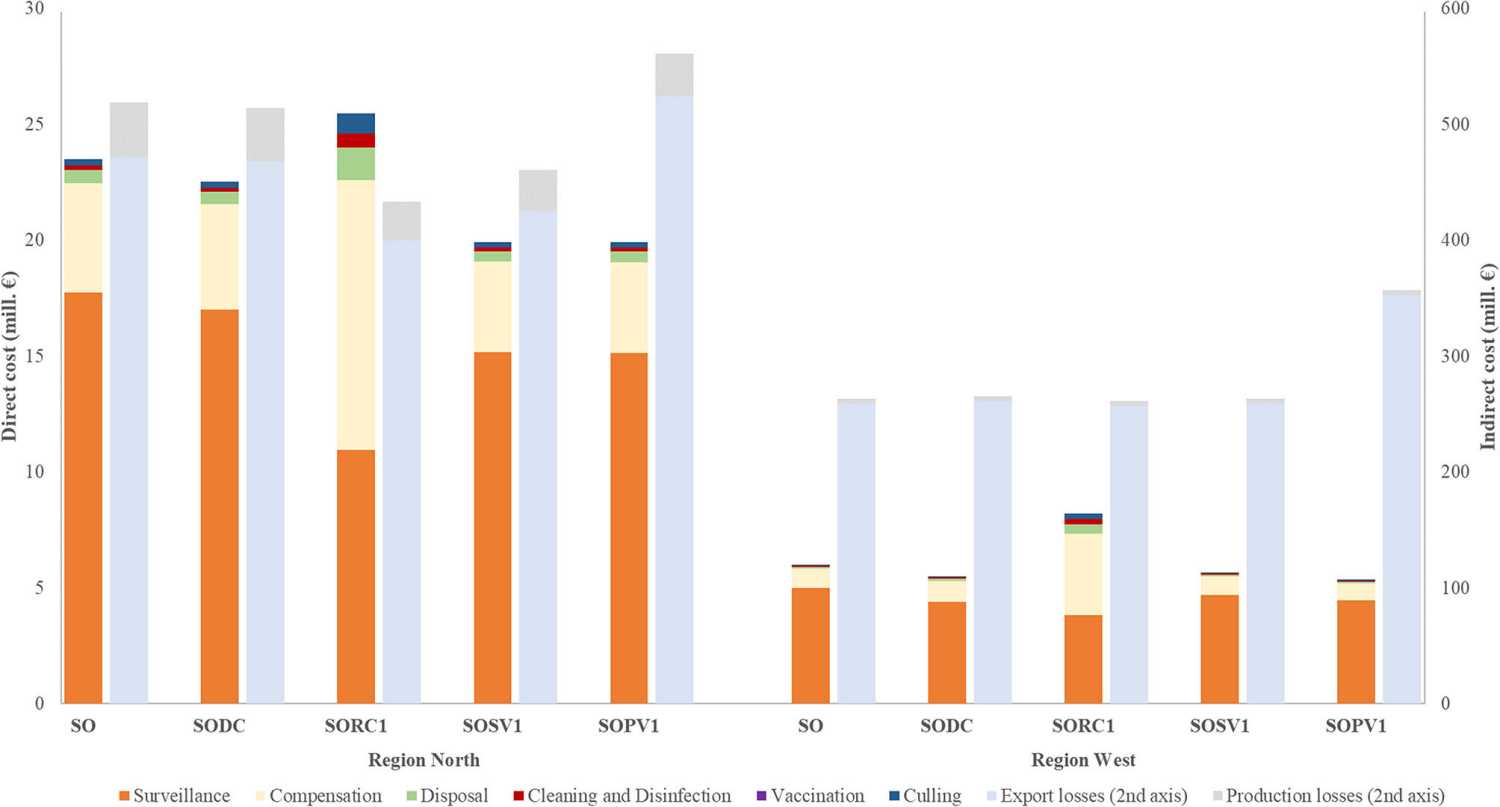
Median direct (1st axis) and indirect cost (2nd axis) incurred due to implemented different control strategies against the FMD outbreaks initiated in “*North*” and “*West*” regions. Direct cost consisted of cost for surveillance, control centers, depopulation, culling, cleaning and disinfection, compensation, and vaccination. Indirect cost included losses from export bans and production losses. The different control measures are: SO, stamping out of all infected herds (reference scenario); SODC, pre-emptive depopulation of dangerous contact herds; SORC1, pre-emptive depopulation of all susceptible herds within 1-km radius around infected herds; SOSV1, suppressive vaccination of all susceptible herds with 1-km radius around infected herds; SOPV1, protective vaccination of all susceptible herds with 1-km radius around infected herds. SODC, SORC1, SOSV1, and SOPV1 are the other additional control measures take into account in the model.

Under the reference simulation scenario the median (5th and 95th percentiles) number of infected farms was 81 (1–427), which corresponds to 0.1% of the total number of FMD-susceptible farms in Austria. The median epidemic control duration (i.e., time from the detection of the first infected farm to the day of lifting of the last restricted zone) was 76 days (23–271) and the median number of depopulated animals was 4,924 (154–28,745) (Figure 2A and Table 3). The total cost for the reference scenario amounted to € 543 (219–1,289) million, of which 4% (1–7%) were direct costs. The largest share of direct costs was allocated to surveillance (74%), followed by compensation (20%) and disposal (2%). The majority of indirect costs (96%) was attributed to export losses (91%), production losses accounted for a share of 9% (Figure 3). All implemented alternative control strategies resulted in fewer infected farms than the reference scenario (Figure 2A and Table 3); the pre-emptive depopulation control strategy resulted in the highest decrease (51%). With the exception of the protective vaccination control strategy, all other alternative scenarios resulted in lower total costs than the reference scenario (Table 3). The implementation of the pre-emptive depopulation control strategy caused the lowest total costs of € 460 (221–853) million (i.e., 15% less compared to the reference scenario; Table 3) and the protective vaccination control strategy resulted in the highest median total costs of € 581 (312–1,043) million. All considered alternative scenarios, with the exception of the dangerous contact herds depopulation strategy were significantly different from the reference scenario (*p* < 0.05), not only in terms of the number of infected farms and the epidemic duration but also in terms of direct costs of the outbreak (Table 3).

**Table 3:** Epidemiological and economic consequences of simulated FMD outbreak in two Austrian regions under different control strategies.

**Scenario^**a**^**	**Infected farms**	**Depopulated farms**	**Depopulated animals**	**Last day of culling**	**Epidemic control duration^**b**^**	**Vaccinated herds**	**Direct cost (in Mio. €)**	**Indirect cost (in Mio. €)**	**Total cost (in Mio. €)**	**Total cost difference to SO strategy**
**Region “*****North*****”**										
SO	81 (1–427)	81 (1–427)	4,924 (154–28,745)	80 (29–189)	76 (23–271)	–	24 (1–95)	519 (217–1,194)	543 (219–1,289)	
SODC	73 (2–380)	76 (3–385)	4,683 (102–27,262)	77 (28–190)	73 (22–244)	–	23 (1–87)	513 (215–1,186)	536 (216–1,273)	−1%
SORC1	40 (3–174)	284 (13–386)	15,422 (503–63,344)	57 (31–101)	150 (25–344)	–	26 (2–102)	433 (219–751)	460 (221–853)	−15%^c^
SOSV1	66 (3–304)	66 (3–304)	4,136 (102–22,380)	69 (29–140)	63 (23–197)	169 (3–760)^d^	20 (1–70)	461 (217–874)	481 (218–944)	−11%^c^
SOPV1	68 (3–323)	68 (3–323)	4,126 (103–22,341)	68 (28–139)	63 (22–204)	171 (3–792)	20 (1–73)	561 (311–970)	581 (312–1,043)	+7%
**Region “*****West*****”**										
SO	15 (1–111)	15 (1–111)	843 (1–7,357)	45 (23–99)	39 (17–96)	–	6 (1–33)	263 (208–392)	269 (209–426)	
SODC	12 (1–79)	18 (1–145)	932 (1–9,486)	44 (23–93)	38 (17–108)	–	6 (1–33)	265 (208–394)	271 (209–424)	+1%
SORC1	12 (1–60)	110 (1–557)	4,051 (55–30,413)	42 (23–72)	62 (17–270)	–	8 (1–43)	262 (208–351)	270 (209–394)	0%
SOSV1	14 (1–98)	14 (1–98)	797 (10–6,267)	45 (23–82)	39 (17–78)	62 (0–339)^d^	6 (1–28)	263 (208–352)	269 (209–380)	0%
SOPV1	14 (1–98)	14 (1–98)	762 (1–6,671)	44 (23–89)	38 (17–75)	63 (0–354)	6 (1–30)	357 (304–461)	362 (305–490)	+35%

Epidemics per region were started in the same 1,000 dairy cattle herds for each control strategy and the model results are presented as median (5th and 95th percentiles).

a The different control measures are: SO, stamping out of all infected herds (reference scenario); SODC, pre-emptive depopulation of dangerous contact herds; SORC1, pre-emptive depopulation of all susceptible herds within 1-km radius around infected herds; SOSV1, suppressive vaccination of all susceptible herds with 1-km radius around infected herds; SOPV1, protective vaccination of all susceptible herds with 1-km radius around infected herds. SODC, SORC1, SOSV1, and SOPV1 are the other additional control measures take into account in the model.

b Epidemic control duration is calculated from the detection of the first infected herd (day 21) to the day of lifting of the last restricted zone.

c Control strategy which compared to reference scenario resulted in significant decrease (p < 0.05) in the number of infected herds, control duration and total cost.

d *Suppressive vaccinated animals will be subject of slaughter once the epidemic is controlled and time and resources permit*.

Depending on the implemented alternative control strategy, export losses caused between 87 and 90% of the total cost, 3–6% of the total costs were allocated to control of the disease and 6–9% to production losses. Figure 3 shows that the highest production losses were incurred within the reference scenario and the dangerous contact herds control strategy, due to the highest number of farms in restricted zones.

The sensitivity analysis demonstrated that variations in the duration of the silent phase have a large impact on the considered output parameters. Decreasing the silent phase of the reference scenario by 7 days decreased the number of infected farms by 70% and control cost by 65%, increasing the silent phase by 7 days lead to a 258% increase of infected farms and 182% increase of control cost. The model was robust to changes in the length of national standstill. Varying the capacity of resources for surveillance had a substantial effect on the outbreak size and epidemic control duration, as determined by the speed with which intervention measures were applied. An increase of the teams by 25% resulted in a 11%-decrease in the number of infected herds, an increase of the teams by 50% in a 26%-decrease, respectively. Changes in the number of teams for cleaning and disinfection under the pre-emptive depopulation control strategy influenced the epidemic control duration but did not reduce the outbreak size (Table 4).

**Table 4:** Sensitivity analysis on resources for surveillance and cleaning and disinfection.

	**Stamping out of all infected herds, reference scenario (SO)**			**Pre-emptive depopulation of susceptible herds (SORC1)**		
	**Reference (surveillance: 160 herds/day; disinfection: 14 herds/week)^**a**^**	**Surveillance +25%^**b**^ (surveillance: 200 herds/day; disinfection: 14 herds/week)^**a**^**	**Surveillance +50%^**b**^ (surveillance: 240 herds/day; disinfection: 14 herds/week)^**a**^**	**Depopulation (1 km radius) (surveillance: 160 herds/day; disinfection: 14 herds/week)^**a**^**	**Cleaning and disinfection +25%^**c**^ (surveillance: 160 herds/day; disinfection: 18 herds/week)^**a**^**	**Cleaning and disinfection +50%^**c**^ (surveillance: 160 herds/day; disinfection: 23 herds/week)^**a**^**
**Region** “***North***”						
Number of infected farms	81 (1–427)	72 (3–351)	60 (2–316)	40 (3–174)	43 (2–160)^e^	44 (3–173)^e^
Epidemic control duration (days)	76 (23–271)	67 (22–226)	59 (21–204)	150 (23–344)	126 (24–344)	104 (25–344)
Cost of control (in Mio. €)	24 (1–95)	24 (1–86)^d^	23 (1–84)^d^	26 (2–102)	27 (1–93)^e^	27 (2–96)^e^
**Region** “***West***”						
Number of infected farms	15 (1–111)	15 (1–116)^d^	14 (1–110)^d^	12 (1–60)	13 (1–66)	11 (1–64)^e^
Epidemic control duration (days)	39 (17–96)	38 (17–105)^d^	36 (17–94)^d^	62 (17–270)	52 (17–214)	41 (17–165)
Cost of control (in Mio. €)	6 (1–33)	7 (1–37)^d^	6 (1–38)^d^	8 (1–43)	9 (1–44)^e^	8 (1–43)^e^

Simulations for sensitivity analysis were run with the reference scenario (to compare the influence of varying the surveillance capacity) and depopulation control strategy (to compare the changes in cleaning and disinfection resources). In both regions, epidemics were initiated in the same 1,000 randomly chosen herds as in previous simulations and the results are presented as median (5th and 95th percentiles).

a The presented capacities of control activities refer to dairy cattle herds, differences in the capacities in other herd types were considered in the calculation.

b The reference number of maximum surveillance visits per day was increased by 25 and 50%, respectively in the sensitivity analysis, all other parameters remained unchanged.

c The reference number of maximum farms which can be cleaned and disinfected per week was increased by 25 and 50%, respectively in the sensitivity analysis, all other parameters remained unchanged.

d Results were not sensitive to changes in resources for surveillance compared to reference scenario.

e *Results were not sensitive to changes in resources for cleaning and disinfection compared to depopulation control scenario*.

### Region “*West*”

Epidemiological results showed that the magnitude of the outbreak in the “*West*” region would most likely be relatively small and of short duration. Thus, the control measures in the reference scenario would be sufficient for bringing the outbreak under control in this region. The reference scenario resulted in 15 (1–111) infected farms, 843 (1–7,357) culled animals and an epidemic control duration of 39 days (17–96) (Figure 2B and Table 3). Total costs amounted to € 269 (209–426) million, of which 2% (<1–8%) were direct costs. Overall, 98% of indirect costs were attributed to export losses.

The suppressive vaccination control strategy showed very similar results as the reference scenario regarding the number of infected farms and total costs (Figures 2B, 3 and Table 3). Slaughter of dangerous contact herds and the pre-emptive depopulation control strategy significantly decreased the number of infected herds. Neither vaccination strategy was significantly different from the reference scenario regarding the number of infected herds, epidemic control duration or the costs of control (Table 3). Considering all alternative control strategies, export losses reached between 95 and 98% of total losses, followed by 2–3% of control cost (Figure 3).

Analogous to region “*North*,” the most sensitive variable was the day of detection. Decreasing the silent phase of reference scenario by 7 days lead to a reduction of number of infected farms by 50% and increasing by 7 days increased the number of infected farms by 80%. The model was sensitive to changes in the capacity of resources for cleaning and disinfection. An increase of the number of available teams by 25% resulted in a 17%-decrease in the epidemic control duration in the depopulation control strategy. An increase of the available number of teams by 50% resulted in a 30%-decrease (Table 4). The model was not sensitive to changes in the number of surveillance teams and the length of the national standstill.

### Resources

The reference scenario analysis of the resources available for performing operation activities in infected farms showed that the number of surveilled farms in the “*North*” region exceeded surveillance capacity immediately after detection day (day 23) and lasted for 41 (5–144) days. In this period, the median number of daily pending surveillance visits was 411 (20–2,762). Analogously, in the “*West*” region the surveillance teams' capacity was exceeded from day 23 for a period of 18 (4–57) days. During this period, 139 (21–868) surveillance visits were pending. We also observed that cleaning and disinfection resources were not sufficiently available for depopulating infected farms in the “*North*” region. A delay of 114 (21–264) days was detected between the day when last animal was culled and the day when the last restricted zone was lifted.

## Discussion

In the current study, we used the EuFMDiS model to simulate the epidemiological and economic impact of an FMD outbreak in two Austrian regions. The model results showed that the epidemiological and economic impact of an FMD outbreak strongly depends on the types of control measures chosen, geographical location of the initial outbreak and the availability of resources to control the outbreak. Comparison of the simulation results between the reference scenario (baseline control measures recommended by the EU) and additional control measures indicated that implementation of further control measures would be more efficient if the epidemic started in an area with high livestock density. The epidemic in the “*North*” region was typically of a large magnitude, driven mainly by the high density of large-scale cattle and pig farms. In contrast, the infection only spread to a limited extent in the “*West*” region, due to low contact rates between the small-scale herds.

In particular, pre-emptive depopulation was identified as the most effective and efficient control strategy in the “*North*” region in terms of a reduction of total costs (reduced by 15% compared to the reference scenario) and number of infected farms (decreased by 51%; Table 3). Analogously, in the “*West*” region the same control measure resulted in the lowest number of infected farms, but total costs were slightly higher compared to other additional control strategies. Pre-emptive depopulation has been analyzed as an FMD control measure in a number of simulation studies, the results of which are consistent with our results that this strategy can considerably limit disease spread (1, 3, 14, 22, 36, 37). However, it can be assumed that pre-emptive depopulation of healthy livestock would be met with considerable resistance in Austria on grounds of animal welfare. It would most likely trigger an intensive public debate and meet with low acceptance in society. Another control measure that was evaluated was the vaccination of livestock. Our findings are in line with the results of other studies (3, 14, 22, 38) and showed a reduction in the spread of FMD through vaccination. The results presented here showed that, regardless of the initial infected region, protective vaccination resulted in the highest total costs compared to all other control strategies, mainly due to high export losses (Table 3).

The strength of the study presented here compared to other available studies (1, 2, 14, 17) is that we evaluated the follow-up costs (i.e., indirect costs) of control strategies, which arose from the business interruption of depopulated farms and the movement bans in restriction zones. We assumed, that once a herd was pre-emptively slaughtered and no revenue was gained, the contribution margin (per production category) represented the production losses for the time of business interruption (Supplementary Table 4). Losses due to movement restrictions in zones represented losses from not collecting the raw milk in dairy farms. Thus, in the reference scenario, costs for farmers of € 47 million (i.e., 9% of indirect cost) and € 4 million (i.e., 1.52% of indirect cost) would incur in the “*North*” and “*West*” regions, respectively. Furthermore, the suppressive vaccination control strategy leads to additional costs, which result from slaughtering the vaccinated animals once the epidemic is eradicated. Neither of the available studies which evaluated the economic consequences of a suppressive vaccination control strategy (3, 14, 22, 38) considered the corresponding follow-up costs in their economic assessments. We estimated that slaughtering vaccinated livestock (including culling, compensation, disposal and cleaning, and disinfection of slaughtered herds) and the subsequent business interruption lead to an additional increase of the direct costs for the “*North*” and “*West*” regions by € 9.4 million (i.e., 46% of direct cost) and € 2.7 million (i.e., 47% of direct cost), respectively. Analogously, the indirect costs would increase by € 2 million (i.e., 0.4% of indirect cost) and € 0.5 million (i.e., 0.2% of indirect cost), respectively. One reason for the different coverage of the costs between studies is the differing definition of the epidemic control duration. Some authors specify the epidemic duration as ranging from the detection of the first herd to the detection of the last herd (1) or to the depopulation of the last herd (14). Hiesel et al. (22) defined the duration of the epidemic as the period from the initial infection of the index herd until the lifting of the last restriction zone. Other studies lack the exact definition of the epidemic duration (3, 36, 37). In our study, the epidemic control duration is defined from the detection of the first infected farm to the day of lifting of the last restriction zone. Thus, outbreak control would continue after the detection of the last herd until all infected farms are culled, disposed, disinfected, and the restriction zones are lifted. This resulted in further costs of control, business interruption, and further demand on resources, which were assessed in the present study.

The analyses of the resources required for operational activities in our simulations showed, that the available resources for surveillance and cleaning and disinfection would not be sufficient to respond appropriately to an FMD outbreak in Austria. Due to insufficient resources for cleaning and disinfection of infected and pre-emptively culled herds in both Austrian regions, the epidemic control duration was approximately extended by a factor two [e.g., in the “*North*” region: last herd was culled on day (median (5th and 95th percentiles)) 57 (31–101) and the last herd was resolved on day (median (5th and 95th percentiles)) 171 (46–365)] compared to the reference scenario. The number of teams needed for surveillance visits also influenced our results substantially. This observation is in line with findings of Garner et al. (39) and Boklund et al. (40). The number of herds to be visited for surveillance in the reference scenarios of both regions exceeded the number of available teams almost immediately after the start of the control phase. This resulted in pending surveillance visits in the simulations. Our findings indicate that in the “*North*” region, an increase in the number of surveillance teams by 50% compared to the reference scenario would reduce the number of infected farms by 25% (Table 4). This leads to a reduction in the magnitude of the outbreak and an increased speed of intervention. In terms of efficiency, this is comparable with additional control strategies such as suppressive or protective vaccination, while maintaining the same resources.

Export losses accounted for the largest share (between 87 and 98%) of total cost associated with an FMD outbreak in Austria. The amount of these costs is substantially determined by the assumption of the export ban duration. In our calculations, we distinguished between EU and non-EU exports and assumed that the exports will resume to EU countries 3 months after culling the last infected herd and to non-EU countries 6 months after culling the last infected herd. We based our assumption on the period after which the status of being free from FMD can be regained and followed the EU council directive 2003/85/EC (29) for EU countries and the Terrestrial Animal Health Code from the OIE (41) for non-EU countries. Our calculations showed that each day of the epidemic (time between detection and culling of the last infected herd) cost Austria € 9–16 million. These costs are in line with the results of Boklund et al., who concluded that even the smallest FMD epidemics with an outbreak duration of 1–3 weeks would cost Denmark between € 340 and 400 million, 97–98% of which would be attributed to export losses. N.B. In the year of publication, Denmark exports were 1.6 times higher than Austrian exports (14). The high total daily losses for Austria show the importance of implementing rapid and effective control measures to reduce the negative economic impact on international trade. Based on our findings, we recommend that the Austrian government should prepare an adequate availability of resources for control activities in order to allow rapid interventions and to minimize the epidemiological and economic consequences of disease outbreaks.

A limitation of our analysis is that the model results represent a “worst-case scenario.” Infections were initiated in early autumn, when the most favorable conditions for airborne dispersion of the FMD virus are present (42) and started in randomly chosen commercial dairy cattle herds, which have the highest probability of infection and spread of the disease. In the EuFMDiS model, dairy herds are parametrized with the highest contact rates compared to other considered herd types. To illustrate the influence of the herd type and regional characteristics, we initiated the simulation in a region with low animal density, such as the “*West*” region, using a detection day of 21, and a randomly chosen index herd (all herd types except dairy herds). The epidemic did not spread beyond the index herd in ~15% of the simulation iterations (results not shown here). The main reason is that the majority of herds in the western part of the country are small, with <15 livestock per herd. Consequently, FMD would be self-limiting and burn out without spreading by the detection day. Another limitation of our study is that topographical features were not considered in the airborne spread pathway of the EuFMDiS model. Approximately 60% of Austrian territory is covered by mountains, the majority of which is located in the western part of the country (region “West”). According to Donaldson and Alexandersen (43) it is expected that the effect of topographical conditions, such as hills and mountains, would cause a plume to deviate and thus reduce the distance of transmission. However, it must be stated, that during a simulation, disease spread became highly localized. About 80% of infections occurred via local spread in region “North” and 60% in region “West,” followed by indirect and direct spread. During the 2001 FMD outbreak in the UK, about 50% of infections occurred within 3 km radius of an infected herd and about 80% occurred within 10 km radius (44). Thus, we do not expect that the lack of topographical features in the airborne spread modeling would influence our results significantly. However, since EuFMDiS also simulates spread of FMD between several European countries with different geographical conditions it would be necessary to consider this variation in the modeling in the future. A further limitation of our study is that the model depends on estimations and assumptions. For instance, the availability of resources (e.g., the number of available surveillance and disinfection and cleaning teams) to respond to an FMD outbreak in Austria is based on opinions of veterinary public health authorities. Uncertainty in such model input parameters can lead to an under-and/or overestimation of our model results, as shown in our evaluation of resource capacities (see: sensitivity analysis). The pre-defined detection day (21 days after initiating the infection) was chosen based on the experience from several recent FMD epidemics (4, 31) and simulation studies (1, 2, 14, 22). Our analysis showed that the parameter “pre-defined detection day” is the most influential factor for the outbreak magnitude. This observations is consistent with findings of other studies (14, 22). Due to differences in the input parameters, geographical and country specific conditions such as livestock structure and the epidemiological situation in the countries such as (non)endemic circulation of FMD virus (45, 46), it is however difficult to directly compare the results of our study with the outputs of other studies.

Furthermore, the losses presented for the Austrian economy due to the FMD outbreak are overestimated because EU-co-financing was not considered. According to EU regulation 652/2015, the EU compensates 50% of the costs for control measures such as destruction of animals and their products, disposal, cleaning and disinfection of herds, and the destruction of contaminated feed (47). Costs for surveillance (including diagnostics) are not subject of co-financing. Thus, the EU would cover ~0.2–1.7% of the total costs in Austria. Furthermore, the presented costs are underestimated because welfare slaughter within restricted zones, which mainly affect pigs and piglets and the costs of destruction of potentially infected feed were not taken into account. Furthermore, the assumption of zoning (zones of export bans) for the intra-community trade lead to a substantial reduction of economic losses in our economic assessment.

The results of the simulations cannot be considered as an exact representation of reality, but they give a range of the expected magnitude of an FMD outbreak in Austria. In general, it is important that decision makers, who use the outcomes of simulation modeling, understand both, the limitations and strengths of these models (12). The realism of data-driven models such as EuFMDiS hinges on the availability and quality of the underlying data. This includes population data, contact structures, environmental data, and pathogen data. Where no data is available, expert opinion can be utilized but this has the potential to introduce uncertainty into a model (24). Simulation models are further limited by the fact that they represent only an approximation of the considered system (12). The elements of the system can only be studied with reasonable effort, when a simplification of the system takes place (13). In contrast, the strengths of disease modeling result from the fact that model outcomes can provide new insights for decision makers. Disease models are ideally able to predict the size and location of the epidemic and they can be used to extrapolate, using the known dynamics for one set of parameters to construct the probable dynamics for another. Models also serve to test rapidly a wide range of control strategies and outbreak scenarios without any of the risk associated with testing during a real outbreak (12).

Veterinary authorities are obliged to take into account various factors when deciding on a mitigation strategy in responding to the incursion of animal diseases such as FMD. This includes the choice of effective control measures, assessing their trade and economic impact, planning, and management of adequate resources and animal welfare. We demonstrated that the choice of control strategy should take into account the characteristics of the affected region and the adequate planning of resources beforehand to effectively and efficiently control an FMD outbreak.

## Conclusions

An outbreak of FMD in Austria would cause total costs between € 269 and 581 million. Our model showed that the epidemiological and economic impact of an FMD outbreak strongly depend on the chosen control measures, the geographical location of the initial outbreak and the availability of resources to control the outbreak. Implementation of additional control measures, according to the EU legislation, would be more efficient if the epidemic started in an area with high livestock density. For instance, for epidemics in areas with high livestock density, pre-emptive depopulation of livestock within a 1-km radius around the infected herds would be the most cost-effective mitigation strategy. In a sparse region, the stamping-out policy of infected herds would be enough to bring the outbreak under control. Adequately increasing resources limits the epidemic magnitude to a degree comparable to additional control strategies such as suppressive or protective vaccination while maintaining the same level of resources.

## Data Availability Statement

The original contributions presented in the study are included in the article/Supplementary Materials, further inquiries can be directed to the corresponding author/s.

## Author Contributions

TM and BP conceived, designed, and coordinated the study. IK and SS parameterized the model for Austria. TM ran the model and summarized the results. TM, BP, and IK drafted and revised the manuscript critically. All authors contributed to the article and approved the submitted version of the manuscript.

## Conflict of Interest

The authors declare that the research was conducted in the absence of any commercial or financial relationships that could be construed as a potential conflict of interest.
