# Supplementary material for: The Epidemiological and Economic Impact of a Potential Foot-and-Mouth Disease Outbreak in Austria
Source: Front Vet Sci. 2021 Jan 13;7:594753. doi: 10.3389/fvets.2020.594753 (PMC7838521; doi:10.3389/fvets.2020.594753)
Supplement: Supplementary file 1 [file Data_Sheet_1.docx]

Supplementary Material

# Supplementary Figures and Tables

## Supplementary Figures

**Supplementary Figure S1.** Visualisation of outbreak spread in the EuFMDiS model. Different colour lines represent the different transmission pathways for the spread of FMD between herds (yellow: indirect infections; red: direct infections; green: local infections; brown (not displayed here): infections via assembly centres; turquoise (not displayed here): airborne infections. Additional panels display following (from left bottom): (i) infection curve; (ii) resources monitor; (iii) operational activities monitor; (iv) vaccination monitor; (v) surveillance monitor and (vi) surveillance queue monitor.

## Supplementary Tables

**Supplementary Table S1.** Time required for specific control activities and maximum number of teams available to manage an outbreak of FMD in Austria.

|  | **Surveillance** | **Culling** | **Disposal** | **Cleaning and disinfection** | **Vaccination** |
| --- | --- | --- | --- | --- | --- |
| **Herd type** | Duration of control activity in days | | | | |
| Large commercial dairy cattle | 0.5 | 1.0 | 0.5 | 1.5 | 0.4 |
| Large commercial beef cattle | 0.5 | 1.0 | 0.5 | 1.5 | 0.4 |
| Small commercial beef cattle | 0.4 | 0.5 | 0.3 | 1.0 | 0.3 |
| Commercial small ruminants | 0.4 | 0.3 | 0.3 | 1.0 | 0.4 |
| Large-scale commercial fattening pigs | 0.3 | 1.0 | 0.5 | 1.5 | 0.2 |
| Large-scale commercial breeding pigs | 0.3 | 1.0 | 0.5 | 1.5 | 0.2 |
| Small-scale commercial pig | 0.3 | 0.5 | 0.3 | 1.0 | 0.2 |
| Backyard | 0.3 | 0.3 | 0.2 | 0.5 | 0.2 |
| Maximum number of available teams | 80 | 24 | 12 | 3 | 60 |

N.B. Resources were estimated by experts from national veterinary authorities and are in line with the suggestions by Roche et al. 2014 (1).

**Supplementary Table S2.** Input parameters used for calculating the direct costs (€).

| **Herd Type** | **Surveillance** | **Culling** | **Disposal** | | **Compensation** | **Cleaning and disinfection** | **Vaccination** |
| --- | --- | --- | --- | --- | --- | --- | --- |
|  | **(per herd)** | **(per animal)** | **(per animal)** | | **(per animal)** | **(per herd)** | **(per animal)** |
| Large commercial dairy cattle | 6,600 | 50 | 120 | | 1,050 | 3,500 | 2.5 |
| Large commercial beef cattle | 6,700 | 50 | 130 | | 1,050 | 3,500 | 2.5 |
| Small commercial beef cattle | 3,200 | 50 | 120 | | 1,050 | 1,200 | 2.5 |
| Commercial small ruminants | 4,000 | 50 | 12 | | 100 | 1,200 | 2.5 |
| Large-scale commercial fattening pigs | 10,900 | 50 | 20 | | 130 | 1,500 | 2.5 |
| Large-scale commercial breeding pigs | 11,300 | 50 | 20 | | 130 | 1,500 | 2.5 |
| Small-scale commercial pig | 5,100 | 50 | 20 | | 130 | 1,000 | 2.5 |
| Backyard | 930 | 50 | 50 | | 425 | 1500 | 2.5 |
| Activities of national control center | | | | 4,500/daily | |  | |

N.B. Costs for surveillance, culling, cleaning and disinfection and vaccination were assessed by the Austrian veterinary authorities. Cost for disposal was obtained from Austrian rendering plants and costs for compensation were derived from the Austrian compensation regulation.

**Supplementary Table S3.** Selected input parameters used in EuFMDiS for the FMD outbreak simulation in Austria.

| **Parameter description** | **Value** | **Reference** |
| --- | --- | --- |
| Initial national livestock standstill period in days following confirmation of the first IF^a^ | 3 | (2) |
| Radius of protection zone in km (area where stringent movement restrictions apply around IF) | 3 | (2) |
| Radius of surveillance zone in km (less stringent movement conditions apply) | 10 | (2) |
| Period of time in days, before movement restrictions can be lifted in surveillance zone (after the last outbreak of FMD has been recorded in this zone) | 15 | (2) |
| Duration in days over which backwards and forwards tracing is conducted on an IF | 14 | (3) |
|  |  |  |
| Probability of reporting suspect herds | 0.592 | (3) |
| Effectiveness of direct tracing  Effectiveness of indirect tracing  Indirect contacts per year  Number of animal consignments moving off a typical herd per year  Number of animal consignments moving on a typical herd per year  Consignment size (most likely)  Proportion of animals in a vaccinated herd that achieve immunity | Cattle 100%  Sheep 90%  Pig 99%  Other 97%  Cattle 60%  Sheep 60%  Pig 85%  Other 69%  Region “North”  Large commercial dairy 450 Large commercial beef 280 Small commercial beef 330 Small commercial ruminants 25 Large-scale fattening pigs 70 Large-scale breeding pigs 90 Small-scale pig 60 Backyard 250  Region “West”  Large commercial dairy 450 Large commercial beef 280 Small commercial beef 330 Small commercial ruminants 25 Large-scale fattening pigs 70 Large-scale breeding pigs 90 Small-scale pig 60 Backyard 250  Region “North”  Large commercial dairy 21.3 Large commercial beef 17.5 Small commercial beef 8.7 Small commercial ruminants 6.2 Large-scale fattening pigs 39.8 Large-scale breeding pigs 72.4 Small-scale pig 14.4 Backyard 4.7  Region “West”  Large commercial dairy 34.4 Large commercial beef 29.1 Small commercial beef 13.6 Small commercial ruminants 4.9 Large-scale fattening pigs 78.7 Large-scale breeding pigs 199.3 Small-scale pig 4.8 Backyard 5.8  Region “North”  Large commercial dairy 10.4 Large commercial beef 31.9 Small commercial beef 4.8 Small commercial ruminants 4.8 Large-scale fattening pigs 15.8 Large-scale breeding pigs 7.4 Small-scale pig 3.8 Backyard 8.3  Region “West”  Large commercial dairy 14.4 Large commercial beef 40.1 Small commercial beef 7.4 Small commercial ruminants 3.1 Large-scale fattening pigs 9.8 Large-scale breeding pigs 31.0 Small-scale pig 1.8  Backyard 8.5  Region “North”  Large commercial dairy 1 Large commercial beef 4 Small commercial beef 1 Small commercial ruminants 4 Large-scale fattening pigs 33 Large-scale breeding pigs 25 Small-scale pig 10 Backyard 1  Region “West”  Large commercial dairy 1 Large commercial beef 2 Small commercial beef 1 Small commercial ruminants 4 Large-scale fattening pigs 11 Large-scale breeding pigs 3 Small-scale pig 2  Backyard 1  Cattle 95% Sheep 80% Pig 87% Other 84% | (3)  (3)  (3)  (3)  (3)  (3)  (3)  (3)  (3)  (3)  (3) |
| Days to complete surveillance visit | Large commercial dairy 0.5 Large commercial beef 0.5 Small commercial beef 0.4 Small commercial ruminants 0.4 Large-scale fattening pigs 0.3 Large-scale breeding pigs 0.3 Small-scale pig 0.3 Backyard 0.3 | Estimated by Austrian Agency for Health and Food Safety/ Federal Ministry of Labour, Social Affairs, Health and Consumer Protection |
| Days to complete infected herds operations (culling, disposal, decontamination) | Large commercial dairy 3 Large commercial beef 3 Small commercial beef 2 Small commercial ruminants 2 Large-scale fattening pigs 3 Large-scale breeding pigs 3 Small-scale pig 2 Backyard 1 | Estimated by Austrian Agency for Health and Food Safety/ Federal Ministry of Labour, Social Affairs, Health and Consumer Protection |
| Days to complete vaccination | Large commercial dairy 0.4 Large commercial beef 0.4 Small commercial beef 0.3 Small commercial ruminants 0.4 Large-scale fattening pigs 0.2 Large-scale breeding pigs 0.2 Small-scale pig 0.2 Backyard 0.2 | Estimated by Austrian Agency for Health and Food Safety/ Federal Ministry of Labour, Social Affairs, Health and Consumer Protection |

^a^ IF, infected farms

**Supplementary Table S4.** Input parameters used for calculating the indirect costs (€).

| **Export losses^a^** | | | |  | **Value** | **Source** |
| --- | --- | --- | --- | --- | --- | --- |
|  | Average daily loss of trade to EU countries: | | | |  |  |
|  |  | | Live animals (€) | | 148,978 | (4) |
|  |  | | Livestock products (€) | | 6,260,022 | (4) |
|  | Average daily loss of trade to non-EU countries | | | |  |  |
|  |  | | Live animals (€) | | 87,197 | (4) |
|  |  | | Livestock products (€) | | 963,345 | (4) |
| **Production losses** | | | |  |  |  |
|  | Production losses in zones | | | |  |  |
|  |  | Percentage of lactating cows in a dairy herd | | | 80 | (5) |
|  |  | Average daily milk yield | | | 24.7 | (4) |
|  |  | Price per 1 kg milk (€) | | | 0.4 | (4) |
|  | Production losses in culled herds | | | |  |  |
|  |  | Average daily contribution margin per dairy cow (€) | | | 4.3 | (6) |
|  |  | Average daily contribution margin per beef cattle (€) | | | 0.5 | (6) |
|  |  | Average daily contribution margin per breeding pig (€) | | | 0.1 | (6) |
|  |  | Average daily contribution margin per fattening pig (€) | | | 0.2 | (6) |
|  |  | Average daily contribution margin per small ruminant (€) | | | 1.1 | (6) |

^a^ Export losses presented here refer only to FMD-susceptible livestock.

**References (Supplementary Material)**

1. Roche SE, Garner MG, Wicks RM, East IJ, de Witte K. How do resources influence control measures during a simulated outbreak of foot and mouth disease in Australia? *Prev Vet Med* (2014) **113**: 436-446. doi:10.1016/j.prevetmed.2013.12.003

2. Council of the European Union - Council Directorate 2003/85/EC. *Off J L 306* (2003) Available at: <https://eur-lex.europa.eu/legal-content/EN/TXT/HTML/?uri=CELEX:32003L0085&from=EN> [Accessed Juni 21, 2020].

3. Bradhurst RA, Garner MG (European Commission for the Control of Foot-and-Mouth Disease, FAO, Rome I.EuFMDiS 1.6. (2019)

4. Anonym (Agrarmarkt Austria). Market Report. (2020). Available at: https://www.ama.at/Marktinformationen/Vieh-und-Fleisch/Aussenhandel [Accessed April 25, 2020].

5. Hiesel JA, Kopacka I, Fuchs R, Schobesberger H, Wagner P, Loitsch A, Koefer J. Epidemiological evaluation of different FMD control strategies in two selected regions in Austria. *Berl Munch Tierarztl Wochenschr* (2016) **129**:484–494. doi:10.2376/0005-9366-15098

6. Anonym (The Federal Institute of Agricultural Economics). Data and Facts - Animal Production. (2020) Available at: https://www.agraroekonomik.at/index.php?id=tierproduktion&L=1&K=0 [Accessed April 23, 2020].
